# A Case-Control Association Study of* RANTES (*-28*C*>*G)* Polymorphism as a Risk Factor for Parkinson's Disease in Isparta, Turkey

**DOI:** 10.1155/2016/5042604

**Published:** 2016-12-18

**Authors:** Nilufer Sahin-Calapoglu, Serpil Demirci, Mustafa Calapoglu, Baris Yasar

**Affiliations:** ^1^Department of Medical Biology, Faculty of Medicine, Süleyman Demirel University, Isparta, Turkey; ^2^Department of Neurology, Faculty of Medicine, Süleyman Demirel University, Isparta, Turkey; ^3^Department of Biochemistry, Faculty of Science, Süleyman Demirel University, Isparta, Turkey

## Abstract

*Background*. Recent studies have revealed that inflammatory processes are involved in the pathogenesis of Parkinson's disease (PD). Multiple lines of evidence have suggested that chemokines and their receptors are involved in several neurodegenerative disorders. We have examined whether genetic polymorphisms at the genes encoding chemokines* IL-8 (-251A>T), MCP-1 (-2518A/G)*, and* RANTES (-28C>G)* and chemokine receptors* CCR2 (V64I*) and* CCR5 (-Δ32)* were associated with sporadic PD risk in Isparta, Turkey.* Method*. The pilot case-control association study included 30 PD patients and 60 control subjects, who were all genotyped with PCR-RFLP for the five polymorphisms. Their genotype and haplotype frequencies were compared statistically.* Results*. One SNP* (-28C>G) *in* RANTES *revealed a significant association with PD (*P* (allele) < 0.0001,* p*-trend = 0.0007). The risk allele* (G)* in the homozygous and dominant models (OR = 17.29 and 32.10, 95% CI = 0.86–347.24 and 1.74–591.937, resp.) suggests additional PD risk. The haplotype* TGCAN* from the* IL-8 (-251A>T), MCP-1 (-2518A>G), RANTES (-28C>G), CCR-2 (V64I)*, and* CCR-5 (-Δ32) *has protective effect (OR = 0.08 [CI = 0.01–0.63], *p* = 0.019).* Conclusions*. Our data are the first indication of the role of* RANTES (-28C>G)* in PD risk.

## 1. Introduction

Parkinson's disease (PD) affects about 2% of the elderly population and is the second most common neurodegenerative disease after Alzheimer's disease (AD) [1–3]. Clinical manifestations include motor symptoms such as resting tremors, bradykinesia, rigidity, and postural instability as well as nonmotor symptoms such as cognitive decline, depression, olfactory deficits, autonomic dysfunction, and sleep disorders. The pathological process underlying PD is the slow and progressive degeneration and loss of dopaminergic neurons in the substantia nigra projecting to the striatum. It is this loss of dopaminergic neurons that causes most of the motor symptoms. Degeneration is not restricted to nigral dopaminergic neurons but also affects noradrenergic, cholinergic, and serotoninergic neurons as well as neurons in the olfactory bulb and mesenteric system [3]. There are also accumulations and aggregations of misfolded alpha-synucleins. Neuron loss and intracellular alpha-synucleins containing inclusions (Lewy bodies and Lewy neuritis) are the pathological hallmark of PD. Intensive research on the etiopathogenesis of PD is still far from explaining the exact cause of the disease. It is a multifactorial disease with likely genetic and environmental determinants. In a small percentage of PD patients, a definitive link has been shown between specific gene mutations and heritable forms of the disease [4]. Genetic predisposition, environmental toxins, and aging are suggested to be the likely key factors in the initiation and progression of the disease [5]. Oxidative stress, mitochondrial dysfunction, excitotoxicity, accumulation of altered proteins, and apoptosis have been implicated as cellular and molecular mechanisms that might be responsible for neuronal degeneration in PD [3]. Results of postmortem and in vivo studies in patients and studies in animal models suggest that neuroinflammation might also contribute to neuronal degeneration [3–10]. The following symptoms in PD patients indicate that there are neuroinflammatory processes in the affected brain regions: the presence of activated microglial cells, reduced density of astrocytes in the substantia nigra, the presence of cytotoxic T-lymphocytes in the substantia nigra adjacent to blood vessels and dopaminergic neurons, and increased concentrations of tumor necrosis factor-alpha, beta2-microglobulin, transforming growth factor-alpha, transforming growth factor-1beta, interferon gamma, and interleukins-1beta, 6, and 2 in the striatum, serum, or cerebrospinal fluid [3].

Chemokines are a large family of structurally homologues cytokines that have a role in the mediation and regulation of immune and inflammatory reactions. They are small polypeptides with a molecular weight of 7–15 kDa. Chemokines are classified by the number and location of N-terminal cysteine residues. The two major groups are CC chemokines (beta-chemokines), in which the cysteine residues are adjacent, and CXC chemokines (alpha-chemokines), in which cysteine residues are separated by one amino acid. The genes encoding CXC chemokines are clustered mainly in the 4q13 chromosome locus, whereas the members of CC chemokines are encoded by genes that are located mainly in the 17q112–12 locus [11]. Chemokines are grouped into two main subfamilies according to their function: inflammatory chemokines control the recruitment of leukocytes in inflammation and tissue injury and homeostatic chemokines fulfill housekeeping functions such as navigating leukocytes to and within secondary lymphoid organs, bone marrow, and thymus during hematopoiesis [12]. During inflammation, CXC chemokines act mainly on neutrophils while CC chemokines act mainly on monocytes, eosinophils, basophils, and lymphocytes [8, 13].

Chemokines, in particular inflammatory cytokines, can exert toxic effects via a direct mechanism, through binding to dopaminergic neurons, or by an indirect mechanism, through glial cell activation and the expression of inflammatory factors [3]. Chemokine receptors are seven transmembrane G-protein coupled receptors that chemokines exert their effects by binding them. Chemokines and receptors are inflammatory mediators with broad potential utility as biomarkers. Chemokines MCP-1 (monocyte chemoattractant protein-1; CCL2) and RANTES (regulated on activation normal T-cell expressed and secreted; CCL5) bind to receptors CCR2 and CCR5, respectively. Constitutive expression of chemokines and their receptors are required for migration, differentiation, and proliferation of glial and neuronal cells. Polymorphisms in the 5′ regulatory region of the genes may be correlated with their levels [14]. In neuroinflammation, increasing evidence of the CCL2-CCR2 and CCL5-CCR5 axes has been demonstrated. Expressional or functional variations caused by genetic polymorphisms of chemokines and their receptors may be associated with the predisposition, pathogenesis, and outcomes of human diseases. Disease-associated genetic variants of* IL-8/CXCL8 (-251A/T), MCP-1/CCL2 (-2518G/A), RANTES/CCL5 (-28C/G), CCR2 (V64I)*, and* CCR5 (-delta32)* have been studied in different populations and ethnic groups with PD and late onset AD [6, 14].

Genetic predisposition has been suggested as an important factor in the etiopathogenesis of PD. In this study, our aim was to assess whether there were genetically driven differences between the immune responses of healthy people and those with PD, specifically in terms of polymorphisms in chemokines and their receptor genes* IL-8, MCP-1, RANTES, CCR2*, and* CCR5*.

## 2. Materials and Methods

### 2.1. Patients and Controls

Thirty patients with clinically definite idiopathic PD, according to the PD Society Brain Bank criteria, were enrolled for the study. All patients were recruited from the outpatient clinic of the Neurology Department, Faculty of Medicine, Isparta Süleyman Demirel University (SDU). Isparta is in southwest Turkey, has a low influence of migration from other countries and interregional movement, and has a population of 230,000 that only 5 percent of them are resettlers.

The average age of disease onset was 62.87 years (range 37–77) and the mean duration of disease was 2.37 years (with a range of 1–4) for PD patients (14 male and 16 female). A total of 60 healthy control subjects (18 male and 42 female) were recruited from relatives or friends of patients in general medical clinics. Their mean age was 60.52 years (range 39–80). Healthy control volunteers and patients were excluded from the study if there were any indications of dementia, stroke, secondary parkinsonism, hypertension, neoplastic or hematological disorders, alcoholism, diabetes, recent infection, hepatic or renal insufficiency, or systemic inflammatory disease.

The study was approved by the Research Ethics Committee of Süleyman Demirel University Hospital (07.15.2015/166) and a written consent statement was signed by all subjects.

### 2.2. Genetic Analyses

A 2 ml blood sample was obtained from patients and control subjects. All DNA samples were obtained using a DNA isolation kit (Thermo Scientific). Genotyping was carried out by the polymerase chain reaction/restriction fragment length polymorphism (PCR-RFLP) method. The PCR-RFLP methods of* IL-8 (-251A>T), MCP-1 (-2518A>G), RANTES (-28C>G), CCR-2 (V64I)*, and* CCR-5 (-Δ32)* polymorphisms were administered, as shown in Table 1.

**Figure 1:**
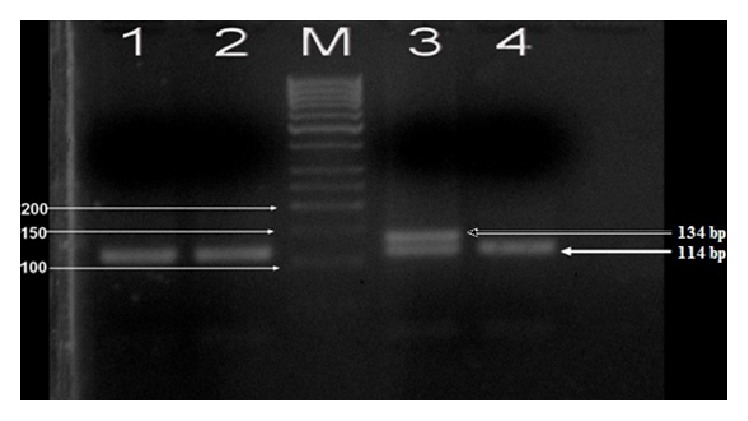
PCR-RFLP agarose gel electrophoresis of* RANTES -28C>G* polymorphism (1, 2, and 4 homozygote normal* (CC)*, 3 heterozygote polymorphic* (CG)*, M: 50 bp DNA marker).

To determine the* RANTES -28C>G *polymorphisms, the relevant area was amplified with PCR and cut with* MnlI *restriction enzymes. Following the application of the PCR-RFLP method for -*28C>G* polymorphism, 114 + 27 + 20 and 13 bp band patterns were observed in normal individuals* (CC)*, 134 + 27 and 13 bp patterns in homozygous individuals* (GG)*, and 134 + 114 + 27 + 20 and 13 bp patterns in heterozygous individuals* (CG)*, respectively (Figure 1).

### 2.3. Statistical Analysis

Genotypes were identified based on electrophoresis banding patterns. Allele and genotype frequencies were calculated by simple allele counting. Genotypes and alleles were expressed as numbers and percentages. Each SNP was analyzed with the FINETTI program (http://ihg.gsf.de/cgi-bin/hw/hwa1.pl) for allele and genotype frequencies (Pearson *χ* ^2^ statistics), odds ratios (OR), and *p* values as well as genetic models such as the homozygous comparison, dominant, recessive, and allele models. When some of the analyzed frequencies were zero, OR was adjusted by Haldane's modification, which adds 0.5 to all cells to accommodate possible zero counts [15]. A Hardy-Weinberg equilibrium of tested groups and Armitage's trend test (ATT) were also calculated using FINETTI. ATT considers genotypes rather than alleles, avoiding a possible bias due to doubling the sample size [16]. The haplotype analysis was performed with the SNPstats program (availability: http://bioinfo.iconcologia.net/SNPstats). All statistical tests were conducted at the 0.05 significance level.

## 3. Results

Genotype and allele frequencies of the relevant polymorphisms were determined from DNA samples of 30 PD patients and 60 control subjects using the PCR-RFLP method. No significant difference was observed between the groups in terms of allele and genotype frequencies of* IL-8 (-251A>T), MCP-1 (-2518A>G), CCR-2 (V64I)*, or* CCR-5 (-Δ32)* polymorphisms. However, there were significant differences for the* RANTES* -*28C>G *polymorphism (Table 2).

The* IL-8 (*-*251A>T)* polymorphism was identified in 63.3% of PD patients and 86.6% of control subjects. Nine patients (30%) were heterozygous for the *T* allele and 10 (33.3%) were homozygous. In the control group, 22 (36.6%) were homozygous and 30 (50%) were heterozygous. There was no statistically significant difference between the patient and control groups in terms of genotype and allele frequency (*p* = 0.107).

The* MCP-1 (*-*2518A>G)* polymorphism was identified in 30% of PD patients and 45% of control subjects. Four PD patients (13.3%) were heterozygous for the *G* allele and 5 (16.6%) were homozygous, while in the control group, 22 (36.67%) were heterozygous and 5 (8.33%) were homozygous. Still, there was no statistically significant difference between the groups (*p* = 0.664).

The* CCR2 (V641) *polymorphism was identified in 23.3% of PD patients and 36.6% of control subjects. Seven (23.3%) PD patients and 22 (36.6%) control subjects were heterozygous for the *I* allele while no homozygous *I* allele was detected in either group. The two groups were similar in terms of* CCR2 (V641) *polymorphism (*p* = 0.201). Neither group included the* CCR5 (-Δ32)* polymorphism.

In terms of the* RANTES -28C>G *polymorphism, 85% of PD patients expressed *C* alleles and the remaining *G* alleles. In the control group, all subjects had C alleles. This allelic difference was found to increase PD risk in our study group at a statistically significant level (OR = 44.456 [CI = 2.540–778.197], *p* = 0.001). Genotype analysis showed the* RANTES -28C>G *polymorphism was present in 12.5% of PD patients but it was absent in the control group. Of the PD patients, 3 (10%) were heterozygous and 3 (10%) were homozygous for the *G* allele. Analysis of possible relationship of genotype frequencies of the* RANTES -28C>G *polymorphism revealed significant differences in the* GG* (homozygous) model compared to* CC* (OR = 17.286 [CI = 0.860–347.237], *p* = 0.009), in the* CG+GG* (dominant) model compared to* CC* (OR = 32, 102 [CI = 1.74–591.937], *p* = 0.0003), and in the* GG* (recessive) model compared to* CC+GG* (OR = 0.065 [CI = 0.003–1.301], *p* = 0.013). The common odds ratio was 4.580 and the *p* value was *p* = 0.0007 in Armitage's trend test. According to the statistical data, the *C* allele of* RANTES -28C>G *polymorphism has a protective effect on the disease and the *G* variant was significantly associated with PD at both allelic and genotypic levels.

Haplotypes with a frequency lower than 5% were excluded from further analysis to minimize loss of power. The most common haplotype was the* A, A, C, G*, and *N* alleles of the* IL-8 (-251A>T), MCP-1 (-2518A>G), RANTES (-28C>G), CCR-2 (V64I)*, and* CCR-5 (−Δ32) *variants. Four haplotypes had a frequency of greater than 5%. The most common haplotype* (A-A-C-G-N)* was used as a reference haplotype in our analysis (Table 3). This was estimated to have a frequency of 0.429. Using this as the reference haplotype, OR was calculated for each possible haplotype. Haplotype 3* (T-G-C-A-N)* was found to be significantly protective for PD (OR = 0.08 [CI = 0.01–0.63], *p* = 0.019). In addition, the global association of haplotype was also found to be significant (*p* < 0.0001) between PD patients and control subjects. None of the other haplotypes were found to be significantly associated with PD (Table 3).

## 4. Discussion

The exact cause of PD is not fully understood. PD's pathogenesis is affected by mechanical and environmental factors, genetic factors, toxins, and oxidative stress. There are no many pathological differences between inherited and sporadic PD. The shared aspects of these forms are the presence of Lewy bodies and the loss of dopaminergic neurons [1, 17].

Chemokines are small peptides involved in the recruitment of leukocytes in inflammation and tissue injury and housekeeping functions in leukocyte trafficking during hematopoiesis [18]. In the central nervous system (CNS), chemokine receptors are expressed primarily by microglia, astrocytes, neurons, and endothelial cells [19]. Among chemokines, interleukin-8 is a key mediator associated with inflammation. It plays a key role in neutrophil recruitment and neutrophil degranulation. Interleukin-8 secretion is increased by oxidant stress, which in turn causes the recruitment of inflammatory cells and induces a further increase in oxidant stress mediators, which is already present in dopaminergic neurons in substantia nigra.

Regarding* IL-8 (-251A>T)* polymorphism, we found no significant difference between PD patients and control subjects. Ross et al. (2004) investigated the relationship of proinflammatory cytokines and Parkinson's disease in an Irish community and found a statistically significant relationship between increased IL-8 levels with* IL-8-251A>T* polymorphism and the disease [20]. They report that the* TT *genotype frequency decreased and* AT* genotype frequency increased significantly in PD patients. However, the difference in genotype frequencies in the PD group did not reach a statistically significant level compared to the control group (*p* = 0.1068). They suggested that the decrease in* TT* genotype, which reduces IL-8 production, might play a protective role against PD. In contrast to Ross et al.'s results,* TT* and* TA* allele frequencies were about the same in PD patients and control subjects in our study (Table 2).

The monocyte chemoattractant protein-1 (MCP-1) is a member of the C-C chemokine family. It is potent chemotactic factor for monocytes. MCP-1 is produced by many cells including astrocytic, monocytic, and microglial cells. Nishimura et al. (2003) reported a relationship between the* MCP-1* -*2518A>G* polymorphism and PD. The* MCP-1* polymorphism was found in 52.5% of 171 patients; 71 patients (42%) were homozygous and 80 (47%) were heterozygous for the *G* allele. It was concluded that -*2518A>G* polymorphism increases the expression of MCP-1 and causes higher microglia activation, which might be effective during the early appearance of PD symptoms [21]. In another neurodegenerative disease, Alzheimer's disease, Pola et al. (2004) showed a relationship with the* MCP-1* polymorphism [22]. However, Huerta et al. (2004) and Gao et al. (2015) found no significant difference in the distribution of* MCP-1* when compared to control subjects [19, 23]. In our study, the difference was not statistically significant, although the percentage of those with* MCP-1* -*2518A>G* polymorphism in the control group outnumbered those in the patient group (Table 2).

CCR2 is a receptor for monocyte chemoattractant factor and mediates monocyte chemotaxis. CCR5 is a beta-chemokine receptor predominantly expressed in T-cells, macrophages, dendritic cells, and microglia. It is crucial for chemokine ligand binding and HIV coreceptor activity. We did not observe any significant difference between PD patients and control subjects for* CCR2* and* CCR5* genotypes (Table 2). Huerta et al. reported similar findings, not only in PD patients but also in Alzheimer's disease patients [19].

RANTES is a selective attractant for memory T-lymphocytes and monocytes and a ligand for CCR5. The NF-*κ*B pathway is known to work in association with the* RANTES* promoter region [24]. RANTES -*403G/A* and -*28C/G* polymorphisms in the promoter region cause a greater expression of RANTES. These* RANTES* polymorphisms were suggested to be effective in several autoimmune diseases [25, 26]. Studies reporting increased serum RANTES levels in association with IL-15, IL-8, and MCP-1 in PD patients suggest that the dysregulation in the peripheral cytokine network might be related to the pathogenesis and underlying neurodegeneration [19, 27, 28]. In addition, RANTES serum levels were reported to correlate with disease severity [29, 30].

In this study, we observed* RANTES -28C>G *polymorphism in 12.5% of PD patients. Of the patient group, 85% expressed *C* alleles and the rest expressed *G* alleles. All the control subjects had *C* alleles. This allele difference in PD patients was found to increase PD risk at a statistically significant level (Table 2). Analysis of possible relationships between genotype frequencies of* RANTES -28C>G *polymorphism revealed that the *C* allele had a protective effect on the disease and that the *G* variant was significantly associated with PD at both allelic and genotypic levels.

Haplotype analyses further confirmed the role of chemokine receptors in PD development. In our study, between-group differences in haplotype frequencies had a global *p* value of <0.0001. The 3-SNP haplotype* T-G-C-A-N* was statistically significant (OR = 0.08 [CI = 0.01–0.63], *p* = 0.019). The frequency of haplotype* T-G-C-A-N* in PD patients (2.28%) was lower than in control subjects (19.2%), suggesting that haplotype* T-G-C-A-N* was statistically related to PD as a protective haplotype.

This is the first study to evaluate* IL-8, MCP-1*,* RANTES, CCR2*, and* CCR5 *polymorphisms in a Turkish population with PD. Our results suggest that the -*28C>G *polymorphism in the promoter region of* RANTES *might increase the protein expression and consequently contribute to the mechanisms underlying inflammation in neurodegeneration.

## Figures and Tables

**Table 1:** Primer sets, melting temperature (MT), PCR product size, and genotyping conditions for *IL-8 (-251A>T), MCP-1 (-2518A>G), RANTES (-28C>G), CCR-2 (V64I)*, and *CCR-5 (-Δ32)* genes as well as single nucleotide polymorphisms (SNPs).

Gene and polymorphism	Primer sets	MT (°C)	PCR product size (bp)	Restriction enzyme	Homozygote normal (bp)	Homozygote polymorphic (bp)
*IL-8* *(−251 A>T)*	F: 5′-GATTCTGCTCTTATGCCTCCA-3′	55°C	816 bç	MfeI	296 + 520	810
	R: 5′-CCCAAGCTTGTGTGCTCTGCTGTC-3′					
*MCP-1* *(-2518 A>G)*	F: 5′-CCGAGATGTTCCCAGCACAG-3′	60°C	930 bç	PvuII	930	708 + 222
	R: 5′-CTGCTTTGCTTGTGCCTCTT-3′					
*RANTES* *(-28C>G)*	F: 5′-ACAGAGACTCGAATTTCCGGA-3′	50°C	187 bç	MnlI	114 + 27 + 20 + 13	134 + 27 + 13
	R: 5′-CCACGTGCTGTCTTGATCCTC-3′					
*CCR-2 (V64I) (+190G/A)*	F: 5′-GGATTGAACAAGGACGCATTTCCCC-3′	63°C	380 bç	FakI	380	215 + 165
	R: 5′-TTGCACATTGCATTCCCAAAGACCC-3′					
*CCR-5* *(-Δ32)*	F: 5′-CCTGGCTGTCGTCCATGCTG-3′	57°C	735 bç	EcoRI	403 + 332	371 + 332
	R: 5′-CTGATCTAGAGCCATGTGCACAACTCT-3′					

**Table 2:** Genotype and allele frequencies of *IL-8 (-251A>T), MCP-1 (-2518A>G), RANTES (-28C>G), CCR-2 (V64I)*, and *CCR-5 (-Δ32)* polymorphisms. Statistically significant values are in bold (HWE: Hardy-Weinberg equilibrium, MAF: minor allele frequency, OR: odds ratio, and 95% CI: 95% confidence interval).

Polymorphism	Groups	Genotypes			HWE	MAF	Association analyses	Genetic model (OR [95% CI]/*p* value)				Armittage's trend test
								Allele	Homozygous	Dominant	Recessive	
					(*p* value)							
		*AA*	*AT*	*TT*				A versus T	AA versus TT	AA versus AT + TT	AA + AT versus TT	Common odds ratio and *p* value
*IL-8* *(-251A/T)*	Case	11	9	10	0.032	0.483	Case versus control	0.582	0.331	0.266	1.158	0,605 *p* = 0.106
	(*n* = 30)	(0,36)	(0,30)	(0,33)				[0.311–1.087]	C.I. = [0.102–1.074]	[0.093–0.760]	[0.460–2.914]	
	Control	8	30	22	0.787	0.617		*p* = 0.088	*p* = 0.061	**p = 0.011**	* p* = 0.755	
	(*n* = 60)	(0,13)	(0,50)	(0,37)								

		*AA*	*AG*	*GG*				A versus G	AA versus GG	AA versus AG + GG	AA + AG versus GG	Common odds ratio and *p* value
*MCP-1 (-2518A/G)*	Case	21	4	5	0.002	0.233	Case versus control	0.837	1.571	0.524	0.455	1.032 *p* = 0.664
	(*n* = 30)	(0,70)	(0,13)	(0,17)				[0.406–1.723]	[0.405–6.092]	[0.206–1.330]	[0.121–1.713]	
	Control	33	22	5	0.740	0.267						
	(*n* = 60)	(0,55)	(0,37)	(0,08)				*p* = 0.629	*p* = 0.511	*p* = 0.171	*p* = 0.236	

		*CC*	*CG*	*GG*				C versus G	CC versus GG	CC versus CG + GG	CC + CG versus GG	Common odds ratio and *p* value
*RANTES (-28C/G)*	Case	24	3	3	0.007	0.150	Case versus control	44.456	17.286	32.102	0.065	4.580 **p = 0.0007**
	(*n* = 30)	(0,80)	(0,10)	(0,10)				[2.540–778.197]	[0.860–347.237]	[1.741–591.937]	[0.003–1.301]	
	Control	60	0	0	0.001	0.000		**p = 0.001**	**p = 0.009**	**p = 0.001**	**p = 0.013**	
	(*n* = 60)	(1,00)	(0,00)	(0,00)								

		*GG*	*GA*	*AA*				G versus A	GG versus AA	GG versus GA + AA	GG + GA versus AA	Common odds ratio and *p* value
*CCR2* (*V64I*) *(+190G/A)*	Case	23	7	0	1.000	0.117	Case versus control	0.588	1.638	0.526	0.504	0.526 *p* = 0.202
	(*n* = 30)	(0,77)	(0,23)	(0,00)				[0.236–1.467]	[0.031–85.372]	[0.194–1.423]	[0.010–26.026]	
	Control	38	22	0	0.185	0.183		*p* = 0.251	*p* = 1.000	*p* = 0.202	*p* = 1.000	
	(*n* = 60)	(0,63)	(0,37)	(0,00)								

		*NN*	*ND*	*DD*				N versus D	NN versus DD	NN versus ND + DD	NN+ ND versus DD	Common odds ratio and *p* value
*CCR5* *(-Δ32)*	Case	30	0	0	0,001	0,000	Case versus control	1.992	1.984	1.984	0.504	nan *p* = nan
	(*n* = 30)	(1,00)	(0,00)	(0,00)				[0.039–101.604]	[0.038–102.405]	[0.038–102.405]	[0.010–26.026]	
	Control	60	0	0	0,001	0,000		*p* = 1.000	*p* = 1.000	*p* = 1.000	*p* = 1.000	
	(*n* = 60)	(1,00)	(0,00)	(0,00)								

**Table 3:** Five marker haplotype estimates and odds ratio (OR) analysis.

Haplotypes	*IL-8*	*MCP-1*	*RANTES*	*CCR2*	*CCR5*	Frequency	OR	*p* value
	*(-251A/T)*	*(-2518A/G)*	*(-28C/G)*	*(V64I) (+190* *G/A)*	*(-Δ32)*		95% CI	
1	A	A	C	G	N	0.429	1.00	—
2	T	A	C	G	N	0.317	0.66 (0.24–1.85)	0.43
3	T	G	C	A	N	0.141	**0.08 (0.01**–**0.63)**	**0.019**
4	T	G	C	G	N	0.065	0.58 (0.10–3.45)	0.55

Global haplotype association *p* value = 0.0001.

PD: Parkinson's disease, OR: odds ratio, and CI: confidence interval.

The most common haplotype is the reference haplotype.

Data are presented as frequencies; *p* values < 0.05 are considered significant.
